# What if… I Asked Cancer Patients About Psychological Distress? Barriers in Psycho-Oncological Screening From the Perspective of Nurses–A Qualitative Analysis

**DOI:** 10.3389/fpsyt.2021.786691

**Published:** 2022-01-26

**Authors:** Lara Dreismann, Alina Goretzki, Viktoria Ginger, Tanja Zimmermann

**Affiliations:** Department of Psychosomatic Medicine and Psychotherapy, Hannover Medical School, Hanover, Germany

**Keywords:** psycho-oncology, screening, interdisciplinary work, distress, cancer nursing, qualitative research

## Abstract

**Introduction:**

Screening questionnaires to assess psychological distress in cancer patients are well-established, but in practice there are difficulties in implementation screening up to referral to psycho-oncology. Interdisciplinary collaboration between psycho-oncology, physicians, and nursing is very important to this process. However, there are barriers and obstacles on all sides.

**Objective:**

The aim of this study is to capture in particular the barriers from the perspective of oncology nursing.

**Materials and Methods:**

Semi-structured interviews with nursing experts (*n* = 15; *n* = 10 female; 24–62 years) from different oncology departments of three university hospitals in Germanys were conducted and qualitative content analysis was carried out by two raters.

**Results:**

The Screening routine is variably well-integrated into daily clinical practice. Structural barriers such as time pressure and a lack of focus on mental distress in nursing are present. Barriers on the side of nurses are primarily a lack of knowledge and communication insecurities when dealing with patients.

**Conclusions:**

There is a need for training and implementation of a disciplinary screening approach. The structural and organizational barriers, which are a challenge for the successful screening process due to unfavorable interdisciplinary team communication and clinical daily structure, should be addressed in further studies. Implications for Practice: In order to establish an interdisciplinary screening process and to overcome the barriers, trainings to deal with knowledge deficits and insecurities seem to be useful.

## Introduction

One in three cancer patients would like psychosocial support (1). Common problems related to the disease are anxiety, worry, and fatigue (2), problems in partnership and sexuality, and challenges in the professional situation accompanied by the risk of unemployment and early retirement (3). In addition, ~50% experience psychological distress, and 30% of patients develop a mental disorder during the course of the disease (4). Regarding psycho-oncological support, however, only 38% feel well-informed (5). Thus, there is a clear need to provide more information about psycho-oncology services and to be able to offer psycho-oncological treatment to patients who need it.

Psycho-oncological screenings have proven to be helpful in identifying these patients in a targeted manner. However, nationwide implementation of psycho-oncological screening of all cancer patients is not yet the norm (6, 7). To better integrate screening for psychological distress into routine clinical practice, international evidence- and consensus-based guidelines provide guidance. In Germany, for example, this is supported by the S3 guideline Psychooncological Diagnosis and Treatment of Cancer Patients (8) and the guidelines of the National Cancer Center Network (9). In particular, brief screening questionnaires to assess psychological distress should be used. The evaluation is based on limit or cut-off values and is inexpensive and less prone to error (10). In addition, computerized screening procedures are also available (11). Moreover, a recent best practice recommendation also addresses guidance on recommended actions for implementing and conducting psycho-oncology screening (12).

Despite the availability of these helpful screening tools, a large proportion of mentally distressed patients are not identified at all or not identified in a timely manner (13). The reasons for the lack of identification of distressed patients are manifold. A number of structural barriers, such as insufficient human and financial resources in inpatient and outpatient treatment settings (10), high workload and time pressure of the medical and nursing team (3, 14), shortened hospital stays (7), and cultural and language barriers (15) are among them. In addition, there is a diffusion of responsibility between medical and nursing teams. In clinical practice, this raises the following questions: Who is responsible for conducting and evaluating the screening questionnaires and initiating psycho-oncological treatment paths? Is it the responsibility of physicians or nursing or case management or the psycho-oncology team. There are different procedures and responsibilities depending on the faculty. In addition, there are doubts in the medical team regarding the acceptably of questionnaire-based screening. Instead, they rely much more on their own clinical experience (16). Unfortunately, few agreements are found between the treatment team's external assessment of psychological distress and the patients' self-assessment (6, 17). A study showed that nurses in oncology centers rated their patients' psychological distress as significantly too low (16). On the other hand, there is a risk that signs of distress (e.g., crying) are overestimated, resulting in misallocation of scarce psycho-oncological resources. The lack of psycho-oncological support for cancer patients is also due to unreliable identification by the treatment team and the lack of a routine screening process (10, 18–20). Thus, needs-based care is not available.

In addition to the aforementioned time and structural barriers, a lack of knowledge and skills regarding the use of screening and lack of confidence are also barriers to conducting psycho-oncological screening (16). At the same time, nurses report that they are quite willing to talk to patients about their psychological distress. However, there is often concern that they are unable to respond appropriately to patients or do not have the necessary communication skills (21). Dealing with mental distress and conducting screenings are often time consuming (22) and involve communication challenges.

Communication skills such as asking open-ended questions, having an empathic attitude, observing non-verbal communication, or active listening are helpful and important in creating an atmosphere of trust between the patient and the oncology treatment team (22). Knowledge of verbal and non-verbal communication can facilitate conversations about patients' psychological distress (23). Communication trainings of the medical team are often very theoretical and not very practice oriented (24). Patients' use of psycho-oncology treatment can only be promoted through communication. The team's recommendation, referral by the treating physician, information about psycho-oncological services, low-threshold access routes, and prior experience with psychosocial support services facilitate patients' use of support services (25).

Despite the aforementioned barriers, identification of patients' psychological distress is significant to prevent negative long-term consequences, low treatment satisfaction, and low quality of life (26). Only through targeted identification and referral to psycho-oncology services that psychological distress, anxiety, depressiveness, and physical symptoms can be decreased and quality of life be improved in the long term (27, 28).

Therefore, the recognition of psychological distress in oncological patients is an important concern in oncology that can only be improved through interdisciplinary work. Known obstacles need to be addressed. Although previous studies have focused on different professional groups with regard to barriers to screening, nurses appear to be a common professional group tasked with screening and are also interested in reducing existing barriers (29). Currently, there is still a lack of clarity regarding barriers and uncertainties on the part of nurses—a professional group that works closely with patients and thus could have good access to patients' psychological distress as well. Unfortunately, there have been only few studies to date that examine the challenges on the part of nurses in more detail. Therefore, the present study focuses on the perspective of the nursing staff. Qualitative interview will be used to identify barriers and challenges of psycho-oncological screening from the nursing perspective. The following research questions will be explored: How do nurses view the current screening process? What barriers exist within the screening process? What barriers are found in providing information about psycho-oncology or referral to psycho-oncology care?

## Materials and Methods

The present qualitative study is an exploratory analysis based on guideline-supported, qualitative expert interviews conducted as part of the multicenter project “OptiScreen—Optimized psycho-oncological care through an interdisciplinary care algorithm—from screening to intervention” funded by the German Cancer Aid (30). A positive ethics vote of the Hannover Medical School is available (No. 8478_BO_K_2019). The presentation of methods and results follows the recommendations of the criteria checklist for reporting qualitative research [consolidated criteria for reporting qualitative research, COREQ (31)].

### Sample

In the period from May to September 2020, *N* = 15 telephone interviews were conducted with experts in the field of oncological care. According to Meuser and Nagel, the corresponding expert knowledge results from the practical performance of certain functions; in this respect, it is specialized expertise (32). The interview participants were selected analogously to a purposive sampling by the nursing service managers of the three study sites (Hanover Medical School, University Cancer Center Leipzig, and University Hospital Carl Gustav Carus Dresden) and through personal contacts with responsible persons on various oncological wards. The study included staff from different nursing areas (inpatient/day-care clinic) and persons with different expertise regarding oncological nursing training [with/without/currently in oncological training, coordinator for oncological training, oncology nursing service (*n* = 4)] and from different medical specialties (visceral oncology, *n* = 12; bone marrow transplantation, *n* = 1; dermatology, *n* = 1; medical case management, *n* = 1; coordinator for education and further training, *n* = 1). In total, *n* = 8 experts were recruited from Hanover, *n* = 3 from Dresden, *n* = 3 from Leipzig, and *n* = 1 additional person was recruited from an external site due to cross-hospital expertise in cross-regional oncology nursing education. There were no dropouts or refusals to participate. Initial contact by e-mail or a face-to-face contact included information about the purpose and procedures of the study, data protection, anonymity, and the voluntary nature of participation. Participants (Table 1) were, on average, 49 years old (SD = 12.83; 24–62), *n* = 10 of them female (67%) and *n* = 5 male (33%).

**Table 1:** Demographic data of the interview participants.

**Variables**	**n**	**%**
Female	10	67
Male	5	33
Mean age (standard deviation, range)	48.67 (12.83; 24–62)	
**Profession**		
Nurse	13	87
Pediatric nursing	2	13
Oncology nursing	10	67
**Work area**		
In-patient	7	47
Day-care clinic	2	13
Out-patient	2	13
Special care advice	3	20
Medical case management	2	13
Others	2	13
Have already received training regarding psycho-oncological/screening	5	33

### Preparation of the Interview Guide

The semistructured guideline used for the interviews was developed on the basis of literature analyses and experiences from everyday work of the psycho-oncology team at Hanover Medical School. The topics and guiding questions were therefore previously defined in a joint workshop. The SPSS method [German: “Sammeln, prüfen, sortieren und subsummieren,” i.e., collect, examine, sort, and subsume (33)] was used to develop the interview guide. This approach is a more widely used procedure for creating guiding questions for interviews. Open-ended questions were used to allow for further additions and new themes from the participants as well. At the same time, these could be clarified through follow-up questions. In addition, quantitative questions were added so that participants could agree or disagree using an 11-point bipolar Likert scale (0 = “disagree at all” / “unimportant” −10 = “fully agree” / “very important”). The interview is divided into introduction, information about the study, informed consent, sociodemographic data, previous experience with psycho-oncological training (e.g., “Have you already attended training on the topic of psycho-oncology in the past, and if so, in what setting and with what context focus?”), current screening procedure in the respective area of work (e.g., “What criteria are used to decide whether psycho-oncological care should be requested?”), current barriers (e.g., “In your view, where might there be barriers to raising the topic of psycho-oncology with patients?”), framework conditions for training (e.g., “In your view, what would be a good time frame for psycho-oncological screening training?”), possible topics for training (e.g., “In your vie, which topics might be neglected or duplicate existing knowledge?”), and concluding remarks. The individual topic sections also formed the basis for subsequent category formation.

### Data Collection

Interviews were conducted by telephone by a psychologist (LD) and lasted between 24 and 79 min (M = 46.60; SD = 15.22). After prior consent from the interview participants, the interview was recorded. Interviewees were alone in a separate room during the phone call to ensure a quiet atmosphere and to reduce face-to-face contact due to coronavirus disease 2019 (COVID-19) hygiene measures. All interviews were pseudonymized and transcribed by a graduate student (AG). Following the concept of data saturation (34), no further interview participants were recruited if neither new nor relevant information was mentioned after 15 interviews.

### Analysis

A rough coding guide was deductively created for the interview data based on the existing themes of the interview guide. To generate categories and structure the material, qualitative content analysis according to Mayring (35) was conducted step by step. Interview material was coded separately by the research assistant (LD) and a graduate student (AG). The intracoder reliability was determined using the Cohen's kappa coefficient, with values of κ < 0.60 considered rather critical and values of κ > 0.75 describing good to very good agreement. In this study, a reliability score of κ = 0.93 was achieved, indicating good to very good agreement. The coding guide was reviewed using the first three interviews (35) and anchor examples, independently coded by project staff, and deductively–inductively supplemented with additional categories or codes and gradually differentiated subcodes to the coding scheme. Individual responses that could not be assigned to a coding were discussed in exchange with each other and recoded (revision of the coding scheme and guideline). Based on the final coding scheme with coding examples and coding rules, further evaluation could take place so that the first interviews could be recoded line by line (35). After completion of the coding phase, the respective categories were analyzed, starting with the superordinate categories and then using the subcategories, to structure the mentioned barriers. Corresponding quotes were compiled to illustrate the themes. The consent questions (Table 2) were analyzed quantitatively.

**Table 2:** Overview of psycho-oncological screening and consultations registration.

**Variables**	**N (%)**
**Screening:**	
Screening questionnaire is used	10 (67)
Screening questionnaire is not used	3 (20)
No information about the use of a screening questionnaire	2 (13)
Medical case management responsible	6 (40)
Nursing staff responsible	2 (13)
Others responsible	2 (13)
**Consultation criteria:**	
Clinical impression	12 (80)
Patient's request	7 (47)
Ticked screening questionnaire	6 (40)
Other criteria	4 (27)
**Consultations registration:**	
Physicians responsible	12 (80)
Nursing staff responsible	6 (40)
Medical case management responsible	2 (13)

## Results

The results identify barriers that complicate the screening process. These are divided into three main categories (screening routine, structural barriers, and barriers on side of nurses) with corresponding subcategories (Table 3). Additional comments by the experts that do not relate to the research questions, such as barriers on part of the patients, are not shown.

**Table 3:** Classification of the interview coding into category systems.

**Main categories**	**Subcategories**
1. Screening routine	
2. Structural barriers	Time pressure
	Short inpatient stay
	No reliable cancer diagnosis
	Focus on surgery
	Not included in the service catalog
	Premises
3. Barriers on the part of nurses	Lack of further training
	Uncertainty in application
	Lack of awareness for psychological stress
	Own load
	Uncertainty about the effectiveness of psycho-oncology
	Psychosocial screening is not defined as an own task
	Differences in the interpersonal expertise of the nurses
	Further barriers on the nurses' part

### Screening Routine

When asked to what extent screening is carried out in the respective medical disciplines and who is responsible for it, the interview participants gave different answers. The majority of the experts (n = 9) stated that screening is carried out using a screening questionnaire. The quantitative agreement question “How important do you think it is to perform a psychosocial screening on every patient using a questionnaire?” (scale from 0 = “unimportant” to 10 = “very important”) showed that interview participants consider screening to be important *(N* = 15; *M* = 9.07; *SD* = 1.34; *Mdn* = 10.00; *Min* = 6; *Max* = 10). Case management (*n* = 6), nursing (*n* = 3), and patient admission management (*n* = 2) were most commonly cited as responsible for conducting the screening questionnaire. Some respondents (*n* = 9) indicated that informational material about psycho-oncology and its various services are available directly to patients or can be distributed to them. According to the interviewees, physicians (*n* = 12) are responsible for registering psycho-oncological consults for patients. The most frequently reported criterion for placing a consil (*n* = 12) is the clinical impression of the patient. Several experts (*n* = 6) reported that this includes, on the one hand, the patients' appearance in the clinic, such as crying or having many uncertainties, questions, or fears about medical treatment (“But they also show outburts of emotion that maybe start crying when you are in the room, and the patients who ask the simplest things are just overwhelmed,” N1208, l. 72), and, on the other hand (*n* = 5), nurses' clinical experience and intuition were also important in identifying a patient's need for psycho-oncological services (“Quite often it is already a gut feeling that you have […].” B0209, l. 52). Respondents also indicated that the patient request (*n* = 7) and completing screening questionnaire (*n* = 6) were also consultation criteria. However, the interviews (*n* = 4) showed that not every department used a screening questionnaire as a screening tool. In addition, other criteria were mentioned, such as routine registration of psycho-oncological consultations after notification of diagnosis (n = 3). It could not be confirmed whether consults are registered regardless of need or only in view of certification (“Psycho-oncology consults are enrolled even if the patient does not have a need in order to meet the “quota” for certification,” *N* = 15; *M* = 2.87; *SD* = 0.99; *Mdn* = 1.00; *Min* = 0; *Max* = 10). Although physicians are responsible for registering consultations (*n* = 12), nurses often (*n* = 5) pass on important information to them or alert them to specific patients with potential needs. According to respondents, this occurs not only during morning rounds (n = 2) but also during the day (*n* = 3). In some interviews (*n* = 3), it also became apparent that the interdisciplinary exchange between the nursing staff and the physicians take place rarely or not at all. This is mainly due to the fact that surgeons are often occupied in the operating room for a long time and are therefore not available as contact persons on the ward. Most of the respondents (*n* = 10) were satisfied with the exchange with the psycho-oncology service. According to the experts, this is because the psycho-oncologists discuss their patients' impressions with the nursing staff before and after a consultation. If this did not occur due to lack of time, the nurses staff could refer to the documentation of the psycho-oncological consultation in the patient's file. In addition, interview participants emphasized the rapid accessibility of the psycho-oncological services in acute situations. Only one department offered a psycho-oncological liaison service, which was rated as more advantageous compared to the consultation service. These advantages included daily care and monitoring of the patient's psychological distress, joint ward rounds, and the possibility of short-term exchange between nursing and psycho-oncology.

### Structural Barriers

Participants mentioned several structural barriers to screening difficult that are not dependent on nursing staff, patients, or the psycho-oncology team but are due to the work environment.

#### Time Pressure

Time pressure during working hours was mentioned most frequently (n = 9) as a barrier by the interviewees. According to the interview participants, nurses focus on treatment care and are unable to adequately assess the mental state of patients due to lack of time. As a result, psycho-oncology is regularly lost in the stress of everyday work. Although the nurses would generally like to approach the patients more often and make psycho-oncology a topic of conversation, they have to set priorities due to the lack of time (“They would like to do that very much because talking to the patients, queries, that is a resource problem, a time resource problem.” W2307, l. 122).

#### Short Inpatient Stay

Interview participants criticized that the time slots for screening were too short due to patients' short length of stay in. In some cases, the registration and realization of psycho-oncological consultations was hardly possible due to the rapid hospital discharge. The experts (*n* = 4) reported that patients sometimes come to the hospital on the day of surgery admission and are discharged within a few days (“[…] and then sometimes (they are) discharged the next day or the day after at latest.” SC1706, l. 42). This problem is exacerbated by a lack of outpatient psycho-oncological offers that might otherwise provide an alternative.

#### No Reliable Cancer Diagnosis

According to the experts (*n* = 5), the short length of stay of patients in hospital often means that a verified cancer diagnosis is not yet available at the time of discharge due to the pending histological findings. The screening questionnaire should only be given to patients with a confirmed cancer diagnosis. Thus, these patients cannot receive psycho-oncological care (“You would actually make an offer (on) the first post-operative day, and on the third, they are already gone or on the second post-operative day, […] if they are still sitting there and do not even know yet whether they have an oncological problem at all.” SC1706, l. 44).

#### Focus on Surgery

Some respondents (*n* = 10) experienced a strong focus on surgery in the surgical ward, so that psychosocial issues were less considered than in non-surgical wards. According to the interview participants, the work environment in abdominal surgery is still underdeveloped in terms of psychosocial stress and less open to psycho-oncology. As a result, respondents reported that it is more difficult for nurses to convince responsible physicians to schedule psycho-oncological consultations. The experts pointed out that surgeons, who are frequently in the operating room and therefore rarely on the ward, only see the patients for short periods of time during the day and therefore cannot properly assess the need for psycho-oncological support (“[…] Surgeons *per se* are very focused on their surgical field. That is really difficult. Also, to push something through for us, we say: “Okay, we would like to have the special oncology care consulting service come or a psycho-oncology consultation as an example.” […]” K2406, l. 90). In addition, adequate and often complex wound care is also a top priority for nurses, so there is usually no time for exploratory and informative discussions with patients.

#### Not Included in the Service Catalog

Some respondents (*n* = 2) noted that neither distribution or evaluation of screening questionnaires nor information about psycho-oncological services is a nursing activity and therefore not mentioned in the service catalog (“For the nursing staff, this is not a core task to collect this screening questionnaire and to have these conversations.” W2307, l. 122). However, the service catalog measures work performance and staffing, so tasks related to psychosocial care were not recorded and therefore not a priority because they could not be documented as a service.

#### Premises

Some respondents (*n* = 5) criticized the lack of space, which resulted in a lack of privacy or a quiet environment in which to have a sensitive conversation about psycho-oncology services. For example, patients could not be found alone, especially in multi-bed rooms, nor would they have the opportunity to retreat when acutely stressed (“[…] then for such conversation, you would actually want to have a quiet environment or so. Not in a hurry.” R2108, l. 310).

### Barriers on the Part of Nurses

From the experts' point of view, there are also personal barriers on the part of the nursing staff that make the use of screening or information about psycho-oncological services difficult.

#### Lack of Further Training

Interview participants (*n* = 8) mostly mentioned a lack of continuing education as a potential barrier in the screening process. Nurses without oncology training lack education and have more difficulty identifying patients with psycho-oncology needs (*n* = 4) (“[…] but the general nurses, who have only completed, in quotation marks, the 3 years of training, are simply not trained well enough. At least for this psychological approach to recognize that […]” N1208, l. 74). In all interviews, a need for further training was evident (“There is perhaps a lack of communication training or something like this.” SM1305, l. 100).

#### Uncertainty in Application

Uncertainty on the part of nurses was frequently assumed to be a barrier by the interview participants (*n* = 6). On the one hand, nurses have difficulties addressing patients appropriately about mental symptoms or offering psycho-oncological services, as they would be fear stigmatization or misunderstanding (*n* = 4) (“Or do I set the right tone in my offer?” B0209, l. 78). On the other hand, they would also fear patient's reaction and not being able to react adequately (*n* = 2), (“That is easy, yes, the fear of the patient's reaction.” W0708, l. 84).

#### Lack of Awareness for Psychological Stress

According to the respondents (*n* = 4), psycho-oncology is often forgotten in everyday ward routine, (“[…] that psycho-oncology is often neglected in my opinion.” B1105, l. 33). Some of the experts (n = 7) saw the reasons for this in a lack of awareness among the nursing staff. Perceiving patients as human beings and not exclusively as persons to be treated seems to be a particular challenge in everyday work, as interview participants mentioned (“[…] the people or patients who come for therapy are not only people to be treated but also people who have a life.” K2605, l. 74). As a result, psychological burden is less considered.

#### Own Load

Nurses' own workload and stress levels were occasionally (*n* = 3) cited as another barrier. As claimed by the respondents, nurses are exhausted because of the constant stress during their work. The nursing staff were claimed to be exhausted due to the constant stress and can only concentrate on the primary care of the patients due to the high intensity of work, the abundance of tasks, and the time pressure. Accordingly, in some cases, the mental state of the patients cannot be adequately addressed (“[…] The nurses on the ward were also somehow dulled.” P2406, l. 50).

#### Uncertainty About the Effectiveness of Psycho-Oncology

Uncertainty about the effectiveness of psycho-oncology was suspected as another barrier (*n* = 3). As interview participants noted, not only some of the patients are biased toward psycho-oncology but also some nurses (“[…] it still has such a negative touch.” SM1305, l. 94). However, if nurses themselves are not convinced about psycho-oncology services and treatment options, it would also be difficult for them to offer these services to cancer patients (“[…] If I have a problem with it, then I cannot sell it to anyone else either […].” SM1305, l. 100).

#### Psychosocial Screening Is Not Defined as an Own Task

In the interviews, it also become clear that some nurses do not see screening, informing, and offering psycho-oncology services as typical tasks of nurses. On wards where only occasionally some oncological patients are treated, these tasks were less established (“[…] Maybe it is not so much focused on […] at the regular ward […]” B0209, l. 74). According to the respondents, it also depends on the importance of psycho-oncology for the nurses themselves, whether they consider it important to ask patients about their mental state or whether they pay special attention to their mental state at all (“But many say to themselves: “Oh, God, yes. I am here to treat and care for him now but not to put up with the psyche.” P2406, l. 80).

#### Differences in the Interpersonal Expertise of the Nurses

Some interview participants (*n* = 2) described the low empathy ability of some nurses as possible barrier, whereby some nurses may not be able to empathize sufficiently with patients and understand their need for psycho-oncological support (“I think that at the wards, there is often a problem of lacking or insufficient empathy […]” SM1305, l. 94). According to the respondents (*n* = 1), some nurses lack the courage and experience to talk empathetically with patients and to inform them about existing offers (“[…] that, of course, you also have to have the courage and perhaps even a certain life experience, how to seek the conversation with the patient, to offer it to him at all.” B0209, l. 76).

#### Further Barriers on the Nurses' Part

In addition, the respondents named further hurdles that could not be assigned to the upper coding categories. According to the interview participants (*n* = 1), some nurses find it difficult to talk to patients because it confronts them with their own psychological burdens or experiences, so this tends to be avoided (“[…] so the more I let it get to me, the more I have to deal with myself.” B1105, l. 77). Furthermore, some interviewed experts (*n* = 2) expressed that some nurses do not carry out the screening because they do not feel that they are taken seriously, are not asked, or do not feel sufficiently involved in the implementation (“And the nursing staff feel, somehow, I don't know, always neglected. So, not seen, not noticed, not perceived […].” B1105 l. 95).

## Discussion

The aim of the present study was to assess experiences with the screening routine and obstacles and barriers with regard to psycho-oncology screening from the perspective of oncology nursing in Germany using qualitative interviews. The results show that from a nursing perspective, there are various structural hurdles and barriers to successfully perform screening or initiate psycho-oncological treatment pathways. The current psycho-oncological care situation was also assessed very heterogeneously. The main outcome categories (screening routine, structural barriers, barriers of side of nurses) go along with the findings of a multidisciplinary interview study that identified barriers in using psycho-oncological services like the lack of organizational and therapeutic integration on the ward, hurdles on the side of the medical team such as personal norms and information deficits regarding psycho-oncology, and hurdles on the part of patients (36).

The current screening routine suggests that criteria for psycho-oncological consults vary. The experts stated different or no experience with screening routine. The existing standards and guidelines of psycho-oncological screening [early and repeated screening using a validated screening questionnaire (8, 9)] seemed unknown by the experts. Hospitals' own guidelines and responsibilities varied as well (e.g., routine daily consultations vs. nurses have to ask physicians to request consultations after screening). This may contribute to structural and personal barriers if responsibility and procedures remain unclear. The structural obstacles largely coincide with existing research findings, such as time pressure and short length of stay. The focus on surgical procedures and somatic treatment, especially in surgery, was emphasized several times (37). Another barrier is that the oncological diagnosis is often not made or communicated during the patient's stay, making inpatient psycho-oncology care impossible (38). Communication in interdisciplinary ward teams is also often inadequate, so that nurses often do not know whether the diagnosis has been communicated to patients. There are studies showing that many nurses are reluctant to express opinions that are contrary to those of the medical team or do not feel sufficiently listened to (39). However, the expertise and clinical impression of nurses are of immense importance. Again, training could be used to strengthen the competencies and role of nurses in relation to screening and to promote exchange about patients' psychological burden.

In addition, the experts emphasized other practical barriers that have received little attention to date. The nursing staff perceives the external conditions, such as rooms and lack of privacy, as inadequate, for example, to address sensitive issues with patients in four-bed rooms. The lack of anchoring in the nursing service catalog makes it difficult to allow time for screenings in everyday life if it cannot be charged through nursing. Once again, a responsibility debate emerges as to who is responsible for screening. One approach to minimize these structural barriers is the use of electronic screening, with automated evaluation and initiation of the psycho-oncological treatment pathway, e.g., in the form of a consult. A recent study demonstrates that direct screening feedback of the result, including a treatment recommendation and query of treatment wishes, leads to an increase in brief contacts to provide information in person (11). This still cannot solve the problem of the lack of importance of psycho-oncology within different disciplines, but it offers a routine implementation into the daily routine of the ward while ensuring the subsequent steps, with explicit involvement of the patients.

From the experts' point of view, the main barriers on the nursing side are the lack of continuing education and training, which is also clearly evident from the literature (21, 40). This goes along with the rather heterogeneous professional experience and different levels of knowledge. However, there is often also uncertainty regarding the indication for psycho-oncological support. Furthermore, some nurses are also unsure whether, when, and why psycho-oncology is effective and helpful. Finally, somatic care is the primary focus, and consideration of psychological issues is sometimes not seen as a scope of duties or its own responsibility. This often results in reduced sensitivity to psychological stress and a lack of awareness of the need for psycho-oncological treatment. In addition, the own (work) burden is a hurdle to actively address screening or psycho-oncology of patients themselves. Another interview study also identified barriers in the area of communication, along with personal barriers such as lack of self-care, anger, and frustration in regard of their own workload and calls for the development of a psychosocial support model for oncology nurses themselves (36, 41).

The only sensible approach to reducing the information deficit and uncertainties in communication is to improve training. This should already be integrated into the training of the nursing professions, at the latest in the oncological specialist training. A recent study (42) describes that the need exists and what additional training might look like. There is already evidence that communication training can alleviate feelings of inadequacy and emotional overwhelm in nurses (43). Both nurses and physicians who have received communication training for oncology patients are more likely to use patient-centered communication and interviewing techniques, which promote the assessment of psychological distress (44, 45). Another study on interdisciplinary collaboration in oncology also sees great opportunities in the education and training of communication skills of different professional groups (46). Another option would be to also systematically involve and train the physicians on the assessment of psychological stress of their patients (47). Following the idea of co-reporting psychological distress together with somatic symptoms, this could promote the importance of psycho-oncological support and integration into the clinical routine of all professional groups (48). Although successful screening alone does not mean actual utilization of psycho-oncology services (49), when screening is combined with a face-to-face conversation and a recommendation or even onward referral, utilization also increases (50). This should be a primary starting point to optimize the identification of psychologically burdened cancer patients and the need-based psycho-oncological care by training medical staff accordingly.

## Limitations

In order to elicit the perspective of nurses, interviews with experts are useful for obtaining a picture of opinions that is close to everyday work–life. However, it must be kept in mind that the insights of the experts cannot be transferred to all nurses, even if they have tried putting themselves in the shoes of their colleagues. It is a limited sample in which the specific professional experience, study sites, specialties, and care settings are heterogeneously distributed. While this allows for the most diverse assessment of the experiences of nursing in oncology, the purposive sampling and the invitation by personal contact might carry a risk of bias. The transfer to other regions, settings, or specialties is not necessarily given. Furthermore, nurses who are particularly sceptical about psycho-oncology or screening would probably not have agreed to participate in the interview.

## Conclusion

Overall, there appear to be several barriers to screening and referral to psycho-oncology from the nursing perspective. Our study was able to uncover those barriers and contribute to a better understanding of the underlying issues. In particular, personal barriers on the part of nurses are a new extension of how screening processes can be improved. To overcome personal inhibitions, uncertainties, and knowledge deficits, specific training of nursing staff could be helpful. Such training should be repeated and address additional barriers and problems encountered during the screening process. It could be adapted to the needs and daily work routine of different settings. Interdisciplinary meetings with the psycho-oncology team could also help to address the aforementioned obstacles, improve exchange, and identify the importance of nurses' perception of patients' distress. In addition, the structural and organizational barriers that pose a challenge to the successful screening process due to unfavorable interdisciplinary team communication and clinical daily structure should also be investigated in further studies.

## Data Availability Statement

The raw data supporting the conclusions of this article will be made available by the authors, without undue reservation.

## Ethics Statement

The studies involving human participants were reviewed and approved by Ethikkommission Hannover Medical School. The patients/participants provided their written informed consent to participate in this study.

## Author Contributions

LD and TZ designed the study. LD conducted the interviews, AG transcribed them. LD and AG coded and analyzed the content. LD wrote the manuscript with support from AG, VG, and TZ. TZ supervised the project. All authors contributed to the article and approved the submitted version.

## Funding

This project was funded by the German Cancer Aid (Deutsche Krebshilfe, 70113550).

## Conflict of Interest

The authors declare that the research was conducted in the absence of any commercial or financial relationships that could be construed as a potential conflict of interest.

## Publisher's Note

All claims expressed in this article are solely those of the authors and do not necessarily represent those of their affiliated organizations, or those of the publisher, the editors and the reviewers. Any product that may be evaluated in this article, or claim that may be made by its manufacturer, is not guaranteed or endorsed by the publisher.
